# Effect of telephone follow-up on repeated suicide attempt in patients discharged from an emergency psychiatry department: a controlled study

**DOI:** 10.1186/s12888-017-1258-6

**Published:** 2017-03-20

**Authors:** Sophie Exbrayat, Clotilde Coudrot, Xavier Gourdon, Aurélia Gay, Jessica Sevos, Jacques Pellet, Béatrice Trombert-Paviot, Catherine Massoubre

**Affiliations:** 1 Department of Emergency Psychiatry, University Hospital, Saint-Étienne, France; 2 Department of Adult Psychiatry, University Hospital, Saint-Étienne, France; 3 Department of Public Health and Medical Information, University Hospital, Saint-Étienne, France; 4 TAPE Laboratory, EA 7423, Jean Monnet University, Saint-Étienne, France; 5 Centre Hospitalier Universitaire de Saint-Étienne, Département de Psychiatrie Adulte, Hôpital Bellevue, Pavillon 52A, 25 Boulevard Pasteur, 42055 Saint-Étienne Cedex 2, France

**Keywords:** Emergency, Follow-up, Nurse, Phone, Suicide attempts

## Abstract

**Background:**

Attempted suicide is a major public health problem, and the efficacies of current postvention protocols vary. We evaluated the effectiveness of telephone follow-up of patients referred to an emergency psychiatric unit for attempted suicide on any further attempt/s over the following year.

**Method:**

In a single-center, controlled study with intent to treat, we evaluated the efficacy of a protocol of telephone follow-up of 436 patients at 8, 30, and 60 days after they were treated for attempted suicide. As controls for comparison, we evaluated patients with similar social and demographic characteristics referred to our emergency psychiatric unit in the year prior to the study who did not receive telephone follow-up after their initial hospitalization. Data were analyzed using logistic regression.

**Results:**

Very early telephone follow-up of our patients effectively reduced recidivism and seemed to be the only protective factor against repeated suicide attempt.

**Conclusions:**

Implementing a protocol of early telephone follow-up after attempted suicide could help prevent repeated attempt/s. More controlled studies are needed to assess optimal techniques to prevent such repetition.

## Background

In 2014, the World Health Organization (WHO) [1] reported that 15 in 100,000 individuals, more than 800,000 each year, die by suicide worldwide, and suicide attempts outnumber deaths from suicide by nearly 20 times. An individual’s history of attempted suicide is predictive of subsequent attempts [2–4], and repeated attempts are considered a risk factor for death from suicide [5, 6]. Suicide and attempted suicide are major public health problems, increasing the costs of healthcare, years of life lost, work stoppages, and emotional burdens on families. The estimated national cost of suicide and suicide attempts was more than $58.4 billion in the United States in 2013 [7] and €10 billion in France in 2009 [8], figures that highlight the need to implement techniques to prevent recidivism. However, studies of the efficacies of various postvention protocols using postcard or telephone follow-up have yielded diverse results [9–16].

France has been ranked 14th in the world for deaths by suicide, with a reported approximately 160,000 people seeking medical attention following a suicide attempt each year [17]. The country’s psychiatric health care is organized geographically on the basis of population size. The territory is divided into sectors, with each sector designated a single medical and paramedical team to address all stages of disease and supervise hospitalization. In our region, most patients who attempt suicide are evaluated in the emergency unit of the university hospital, undergoing physical examination followed by a meeting with a psychiatrist.

### Objectives of the study

In patients treated for attempted suicide in our university hospital’s Department of Emergency Psychiatry, we prospectively evaluated the efficacy of a program of telephone follow-up over the year following the initial visit on any further attempt/s. Study patients included those seen as outpatients as well as those admitted for no more than 3 days. The study was controlled and conducted with intent to treat. Limited resources required that it be a cost-saving program easily implemented and well accepted by patients and teams [18, 19]. A program of very early intervention (at +/− 8 days) was proposed.

## Methods

### Participants

Study patients included those admitted to the Department of Emergency Psychiatry for suicide attempt between 01 January through 31 December 2010 who resided within the catchment area of the hospital, were at least 18 years of age, had no history of psychiatric hospitalization exceeding 72 h in a crisis unit, and whose situation permitted follow-up.

We excluded patients younger than 18 and whose psychiatrist might judge follow-up to be potentially harmful (such as patients with a personality disorder like dependent personality) or whose inclusion might interfere with an established program of allied intensive care. Study patients received usual treatment and telephone follow-up.

For comparison, we reviewed medical records of patients who had attempted suicide and were admitted to our emergency psychiatry department between 01 January 2009 and 30 November 2010, before implementation of this study, and identified controls who fit the same inclusion criteria as those of the study participants. The control patients also received usual treatment at the time of suicide attempt, but they did not receive subsequent telephone follow-up.

Our hospital’s local ethics committee approved the study, and informed written consent was obtained from participants.

### Study setting

At the patient’s initial interview, the consulting psychiatrist used an interview guide to record the date of consultation, name of the patient’s general practitioner, name of the interviewer’s name, administrative data (address, phone number/s, e-mail address, means to contact the patient again if not reached by call, clinical data (date of the suicide attempt, means used for the attempt), medical history, psychiatric history (number of suicide attempts, number of psychiatric hospitalization/s and locations, history of psychiatric disease in the family, serious family events in childhood), current treatment (name of psychologist and/or psychiatrist, prescribed drugs), use of alcohol, cannabis or other substance, marital status, financial, family, and social resources and support, protective factors, such as religious or social activities, recent life events, such as unemployment, conflicts, death/s of someone close, or separation or divorce, current symptoms including changes in mood, sleep, appetite, anxiety, and others noted by the patient, diagnosis according to the International Classification of Diseases (ICD10), such as depression, bipolar disorder, anxiety disorder, schizophrenia, other psychosis, personality disorder, or other diagnosis, decision at the end of the consultation for hospitalization or not, if no hospitalization recommendation after the consultation for follow-up with the patient’s GP, psychiatrist or psychologist***.***

A specially trained nurse then used some recorded data to assess suicide potential according to a 3-point scale described by Shea [20] that rated risk, emergency and degree of harmfulness. At the end of the consultation, the patient was advised that he or she would receive 3 follow-up telephone calls − at 8 ± 2 days, 30 ± 5 days, and 60 ± 5 days after the suicide attempt. The same nurse who attended the inclusion interview called the patient, completed the same 3-point scale assessing suicide risk, emergency and degree of harmfulness and assessed medication compliance.

At each designated time, the nurse called the patient up to 3 times between 1:00 and 8:30 p.m. over the same day, and if there was no answer, the nurse left a message informing the patient of the purpose of the call and asking for news. If the patient answered the initial call or called back, the interview was conducted within 24 h, and suicidal potentialities were assessed. However, if the calls were not returned, a text message, e-mail, or letter was sent within 24 h and followed by a new letter within 7 days of the nurse’s call and then every month thereafter over 5 months if the patients still failed to respond.

When a patient made a subsequent suicidal gesture during the period of follow-up, the protocol of telephone follow-up was restarted from the date of the second attempt without requiring the individual’s new inclusion into the study.

The primary endpoint was the rate of recidivism one year after the initial episode.

In our study, we defined recidivism as a repeated suicidal gesture and obtained those numbers by a systematic review of the electronic medical records of each patient, which included any consultation or hospitalization within the psychiatric sectors depending on our university hospital.

### Statistical analysis

Data were analyzed using statistical analysis software (Version 9.2, SAS Institute Inc., Cary, North Carolina, USA). Chi-squared test was used to assess qualitative variables and t-test, to evaluate quantitative variables, with *P* < .05 considered significant. Multivariate analysis used logistic regression. We analyzed variables that differed significantly between the 2 groups, variables significantly correlated to recidivism in univariate analysis, and partial confounding factors (*P* < .10 in univariate analysis).

## Results

Table 1 shows the sociodemographic characteristics and diagnoses of the 823 study and control patients − 436 in our study group and 387 in the control group. Among the study patients, 56% responded to all 3 follow-up telephone calls and 32.3%, to either one or 2 calls; 11.7% did not respond to any of the calls. The 2 groups did not differ significantly with regard to sex, age, financial resources, social support, employment status, history of psychiatric hospitalization, and diagnoses of psychotic, bipolar, personality, and eating disorders or psychosocial crises. However, those in the control group showed more suicide attempts, treatment with psychotropic drugs, and psychiatric follow-up care than our study patients, who demonstrated significantly more major depressive and anxiety disorders.

**Table 1:** Sociodemographic and psychiatric characteristics for study and control patients

	Study patients *N* = 436 N (%)	Control patients *N* = 387 N (%)	*P*
Sociodemographic characteristics:			
Women	312 (71.6%)	261 (67.4%)	0.199
Men	124 (28.4%)	126 (32.6%)	
Age (years) mean ± standard deviation	40.2 ± 15.3	39.7 ± 15.1	0.661
Social support	56 (12.8%)	53 (13.7%)	0.719
Employed	186 (42.7%)	148 (38.2%)	0.792
Resources	354 (81.2%)	240 (62.0%)	0.193
Psychiatric characteristics:			
Previous suicide attempt	199 (45.6%)	213 (55.0%)	0.0002
Previous psychiatric hospitalization	172 (39.4%)	123 (31.8%)	0.852
Psychotropic treatment	289 (66.3%)	218 (81.7%)	0.0001
Psychiatric follow-up care	148 (33.9%)	146 (37.7%)	0.035
Major depressive disorder	186 (42.7%)	124 (32.0%)	0.027
Bipolar disorder	10 (2.3%)	5 (1.3%)	0.559
Psychotic disorder	18 (4.1%)	21 (5.4%)	0.372
Personality disorder	142 (32.6%)	123 (31.8%)	0.593
Anxiety disorder	40 (9.2%)	12 (3.1%)	0.0005
Eating disorder	4 (0.92%)	4 (1.0%)	0.999
Psychosocial crisis	121 (27.8%)	86 (22.2%)	0.069
Recidivism	55 (12.6%)	69 (17.8%)	0.037

### Rate of repeated suicide attempts

Repeated suicide attempts were significantly fewer among study (55/436) than control (69/387) patients after the initial index episode (*P* = 0.037) (Table 1). Calculation of the rate/ratio showed a drop of 33%***.*** For the 244 patients who responded to all telephone follow-up calls, the OR of recidivism was even lower: 0.50 (95% confidence interval [CI],0.62 to 0.80).

### Factors of recidivism and protective factors

#### Univariate analysis

Table 2 shows that telephone follow-up and diagnoses of psychosocial crisis were protective factors against repeated suicide attempt. Risk factors favoring repeated suicide attempt included previous suicide attempt, previous psychiatric hospitalization, previous treatment with psychotropic drug/s, current psychiatric follow-up care, and presence of personality disorder (see Table 2).

**Table 2:** Factors affecting recidivism of suicide attempt and protective factors

	Univariate analysis			Multivariate analysis Adjusted according to the other variables of the model		
	Odds ratio	95% confidence interval	*P*	Odds ratio	95% confidence interval	*P*
Sociodemographic characteristics						
Age (years)	0.99	0.99 - 1.01	0.842			
Women	1.08	0.71 - 1.64	0.724			
Social support	1.35	0.80 - 2.28	0.256			
Employed	0.75	0.50 - 1.13	0.164			
Resources	1.70	0.90 - 3.21	0.101	2.55	0.84 - 7.78	0.100
Psychiatric characteristics						
Previous suicide attempt	2.77^a^	1.81 - 4.26	<.0001	1.53	0.82 - 2.86	0.186
Previous psychiatric hospitalization	2.66^a^	1.77 - 4.00	<.0001	1.91^a^	1.01 - 3.61	0.046
Psychotropic treatment	5.15^a^	2.45 - 10.8	<.0001	2.74^a^	1.12 - 6.70	0.027
Psychiatric follow-up care	2.61^a^	1.77 - 3.86	<.0001	1.11	0.60 - 2.06	0.737
Major depressive disorder	0.86	0.57 - 1.29	0.459	0.76	0.40 - 1.45	0.408
Bipolar disorder	1.41	0.39 - 5.08	0.597			
Psychotic disorder	1.75	0.81 - 3.78	0.156	1.71	0.55 - 5.30	0.355
Personality disorder	3.11^a^	2.08 - 4.67	<.0001	2.49^a^	1.23 - 5.04	0.012
Anxiety disorder	0.86	0.38- -1.95	0.712	1.38	0.46 - 4.15	0.564
Eating disorder	0.80	0.10 - 6.51	0.830			
Psychosocial crisis	0.30^b^	0.17 - 0.55	<.0001	1.14	0.49 - 2.66	0.769
Telephone follow-up	0.67^b^	0.45 - 0.98	0.034	0.45^b^	0.26 0.78	0.004

^a^risk factor; ^b^protective factor

The interval between the index episode and the first repeated suicide attempt was 143.9 days (± 105.3) in our study group and 107.0 days (± 105.2) in the control group (*P* = 0.05).

**Fig. 1:**
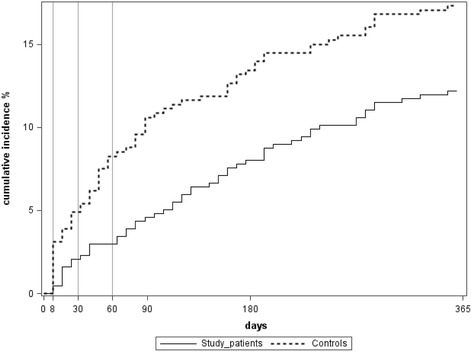
Cumulative incidence of recidivism (log-rank test, *P* = 0.02) during follow-up

Among controls, the cumulative incidence of recidivism grew faster during the period immediately following the initial attempt, at the beginning of the period of telephone follow-up of the study group (Fig. 1).

To specify the effect of each call, the OR of recidivism was calculated for each period following a call (from 8 days to 30 days for the first period, from 30 to 60 days for the second period, and from 60 to 90 days for the third period). These ORs, adjusted on previous suicide attempts were 1.07 (0.40 to 2.82) for the first period, 0.31 (0.10 to 0.98) for the second period, and 0.67 (0.23 to 1.96) for the third period. Only results for the period following the second call (30th day) differed significantly from those of the control group. Age and gender did not influence recidivism according to the period.

#### Multivariate analysis

Multivariate analysis demonstrated only telephone follow-up as a protective factor against repeated suicide attempt (see Table 2), whereas risk factors favoring recidivism included treatment with psychotropic drug/s, previous psychiatric hospitalization, and presence of personality disorder (see Table 2).

The relative risk of recidivism in the subgroup of patients who completed all 3 follow-up telephone interviews compared with that of controls and adjusted for the same factors was 0.41 (95% confidence interval [CI], 0.22 to 0.76).

## Discussion

In this single-center prospective study, both univariate and multivariate analyses demonstrated the efficacy of telephone follow-up of patients as a protective factor against repeated suicide attempt within one year of their initial treatment. These results agree with those of a recent Spanish study on telephone follow-up [21] and particularly with those of a large-scale study of the WHO [1] in 5 countries that showed significantly fewer suicides in their group receiving follow-up calls [22]. In a French study [14] designed similarly to ours, the intent-to-treat analysis showed no efficacy of telephone follow-up, but the per-protocol analysis demonstrated that systematic follow-up by telephone one month after suicide attempt halved the number of repeated suicide attempts during one year of follow-up. Moreover, they reported that in 48 of the 150 cases in which a self-aggressive gesture was repeated, the first follow-up telephone call was made only after the first repetition of suicide attempt [13].

The cumulative incidence curve of recidivism also seems to indicate that the difference between the 2 groups lies mainly in the first weeks following the suicidal gesture. Thus, prompt recontact of patients seems most effective.

Our findings of univariate analysis also agree with those in the literature. Indeed, previous suicide attempt, previous psychiatric hospitalization, and personality disorder are known risk factors that favor repeated suicide attempt [9, 23, 24]. Conversely, psychiatric follow-up, treatment with psychotropic drug/s, and diagnosis of psychosocial crisis (i.e., without major psychiatric disturbance) are protective factors against repeated attempt/s. The increasing interval between the index episode and recidivism also agrees with that reported in the literature.

A number of limitations must be noted. Our protocol planned to recontact people who repeated a suicidal gesture after their initial inclusion event. Motivated by ethical reasons, we did not want to deprive of telephone follow-up patients who repeated a self-aggressive behavior and so chose not to record a repeated event as a separate inclusion.

Several variables differed significantly between our study and control patients. Sample-selection bias may account for the greater number of previous suicide attempt/s and drug treatment among controls than study patients, and the selection of controls from medical records might have led to higher rates of non-inclusion, exclusion, and refusals in the study patients. Similarly, patients presenting with a major depressive episode might have more readily agreed to participate in the program than those without depression, yielding a higher rate of depression among study patients (42.7%) than controls (32.0%), and anxiety disorders not considered as primary diagnoses might be under-reported in the control group.

Psychiatric diagnoses are not highly reliable because they are made after a single consultation in a context of psychological crisis. For this reason, Vaiva and associates [16] matched some patients only according the number of previous suicide attempts.

To take into account the above-mentioned factors, we used a multivariate model, and that analysis suggested a possible relationship between previous suicide attempt/s and hospitalization/s with the criteria for inclusion and exclusion and hospitalization. Similarly, characteristics of the study population, such as suicide attempt without hospitalization, could also play a role [25]. This may reflect a hidden selection bias that favors inclusion. Furthermore, interactions between the different dimensions of psychiatric history and correlation between psychiatric follow-up and treatment could explain the finding as risk factors of previous psychiatric hospitalization and treatment with psychotropic drugs, but not previous psychiatric follow-up care. It is probable, however, that patients with a history of hospitalization and/or psychotropic treatment have also received follow-up care. Still, though treatment implies the person’s need for care, access to drugs could increase the risk for overdose. The same reasoning applies in the case of psychosocial crisis.

We may also have underestimated the rate of recidivism because some patients may have attempted suicide again and been referred to other hospitals. Nevertheless, Kapur and colleagues [26] reported that 80 to 90% of patients who have attempted suicide again are admitted to the same hospital.

Those who attempt suicide are not a homogenous group [27, 28] and are unlikely to respond to a single postvention technique. The use of postcards, for example, has seemed more efficient in women than men [11]. Moreover, telephone follow-up may be inefficient in groups of patients who have attempted suicide for the first time [11, 16]. Very large studies would be required to evaluate various subgroups of patients and postvention techniques, but implementation of such studies would be limited by the sample sizes required to obtain sufficient power.

## Conclusions

Telephone follow-up of outpatients after suicide attempt is a protective factor against repeated suicide attempt/s and possible consequent suicide and could be applied in all psychiatric emergency departments. New studies with more patients and in multiple centers are needed to examine more specific patient populations and postvention techniques***,*** but implementation of such studies could be cost prohibitive.

## Abbreviations

CI: Confidence interval
ICD: International Classification of Diseases
OR: Odds ratio
WHO: World Health Organization
